# Synergistically reinforced bioresorbable hydrogel for mechanical–biological decoupling and osteogenic–angiogenic coupling

**DOI:** 10.1016/j.mtbio.2026.103736

**Published:** 2026-10-13

**Authors:** Yue Hong, Huali Lu, Jie Luo, Yaoyi Li, Yizi Ye, Yuning Hua, Huaizhong Xu, Ting Xu

**Affiliations:** a Department of Stomatology, The First Affiliated Hospital, Zhejiang University School of Medicine, Hangzhou, 310003, PR China; b Department of Biobased Materials Science, Kyoto Institute of Technology, Matsugasaki, Kyoto, 606-8585, Japan

**Keywords:** Melt electrowriting, Reinforcement, Hydrogel, Poly(lactic-co-glycolic acid), Bone regeneration

## Abstract

Ideal hydrogels for craniofacial bone regeneration should combine mechanical robustness, tunable bioresorption, and synergistic osteogenic–angiogenic bioactivity. However, conventional hydrogels suffer from an inherent trade-off between mechanical performance and biological activity, since both properties are dominated by crosslinking density. Here, a synergistically reinforced bioresorbable hydrogel system is developed by integrating highly ordered melt electrowriting poly(lactic-co-glycolic acid) (PLGA) microfibrous scaffolds with bioactive glass (BG)-reinforced and functionalized gelatin methacryloyl (GelMA) matrix. This architectural reinforcement provides a strategy for partial mechanical–biological decoupling, enabling mechanical properties to be tailored independently of hydrogel crosslinking while preserving the bioactivity of the GelMA phase. The composite hydrogel efficiently promotes osteogenic differentiation of rat bone marrow mesenchymal stem cells and enhances angiogenic activities of human umbilical vein endothelial cells. The enrichment of Ras/MAPK signaling suggests a potential bifunctional role of this pathway in osteogenic and angiogenic regulation. In a rat critical-sized calvarial defect model, the scaffold remarkably induces extensive vascularized bone regeneration, achieving a bone volume fraction of 59.9 ± 7.4% at 12 weeks post-implantation. This work establishes a synergistic reinforcement strategy that decouples mechanical and biological functions while orchestrating osteogenic–angiogenic coupling for craniofacial bone regeneration.

## Graphical abstract

**Figure:**
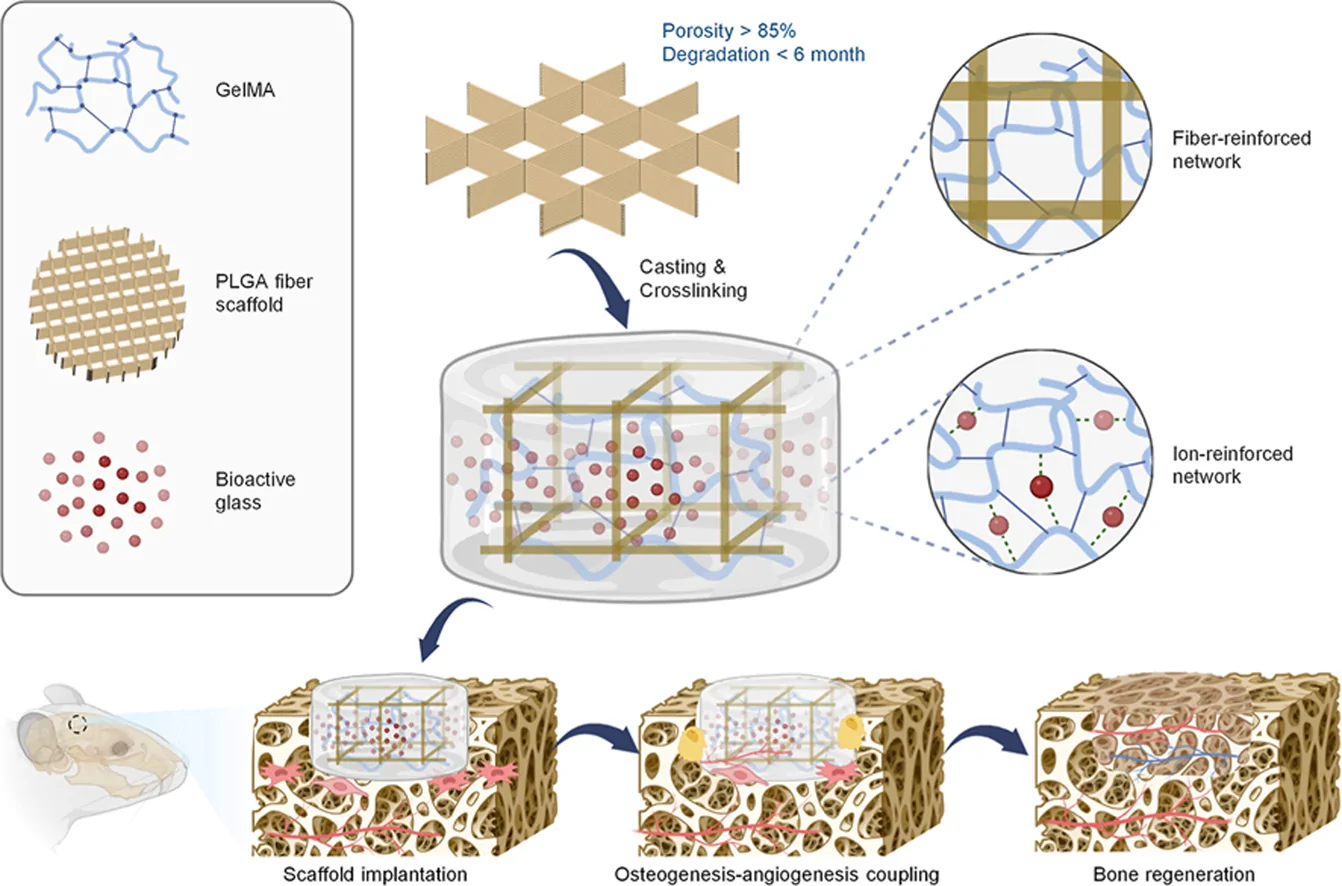

## 1 Introduction

Critical-sized craniofacial bone defects arising from trauma, tumor resection, or congenital anomalies continue to pose significant challenges in reconstructive surgery [1], [2], [3]. Distinct from long bone defects, craniofacial defects exhibit limited intrinsic vascularization and require precise anatomical reconstruction, making spontaneous healing insufficient for functional recovery [4], [5]. Autologous bone grafting is currently regarded as the gold standard for craniofacial reconstruction [6]. However, donor-site morbidity, limited graft availability, and secondary surgical trauma restrict its clinical application. Although allografts and xenografts offer alternative options, concerns regarding immune rejection, disease transmission, and inadequate integration with host tissue continue to limit their clinical efficacy [7], [8]. In this regard, tissue-engineering scaffolds have emerged as a promising alternative for craniofacial bone regeneration [9].

Despite extensive advances in hydrogel-based scaffolds, achieving mechanical robustness, controllable bioresorption, and biological functionality within a single platform remains challenging [10]. In most hydrogel systems, these properties are intrinsically governed by crosslinking density. Increasing crosslinking density generally improves mechanical strength and slows degradation, thereby enhancing structural stability [11], [12]. However, dense polymer networks can restrict cell infiltration, matrix remodeling, nutrient transport, and tissue integration. In addition, changes in photocrosslinking conditions can affect not only the mechanical properties of GelMA hydrogels but also cell-related responses [13], [14]. Studies using fibrous GelMA-based hybrid scaffolds have further shown that increasing the degree of GelMA crosslinking can affect cell spreading [15]. Conversely, reducing crosslinking density can improve cellular responses and biological activity but frequently compromises mechanical integrity and dimensional stability [16]. This intrinsic coupling between crosslinking density and both mechanical and biological properties limits the ability to optimize these functions independently. A strategy that separates mechanical reinforcement from hydrogel crosslinking could therefore provide greater flexibility in balancing structural stability, bioresorption, and biological functionality.

Structural reinforcement through an architected scaffold provides a potential route to reduce the dependence of mechanical performance on hydrogel crosslinking. Melt electrowriting (MEW), a high-resolution additive manufacturing technology, enables the fabrication of highly ordered microfibrous architectures with precisely controlled fiber diameters, pore geometries, and spatial organization [17], [18]. Dubey et al. used MEW-printed poly(caprolactone) (PCL) to reinforce GelMA [19]; similarly, Meng et al. used poly(L-lactic acid) to reinforce gelatin hydrogel [20], demonstrating that incorporating an MEW fibrous framework into bioactive hydrogels can enhance mechanical stability, regulate degradation, and promote osteogenic activity. However, the reinforcing architecture has generally been considered as an additional structural support rather than as an independently adjustable mechanical design parameter. Consequently, whether variations in MEW architecture alone can systematically regulate the mechanical performance of the composite while maintaining an unchanged hydrogel formulation and crosslinking condition remains insufficiently explored.

While structural reinforcement can reduce the dependence of mechanical performance on hydrogel crosslinking, a biologically active matrix is still required to support tissue regeneration. Gelatin methacryloyl (GelMA) has been widely used as a photocrosslinkable hydrogel owing to its excellent cytocompatibility and extracellular matrix-mimicking characteristics [11], [21], [22]. However, GelMA alone exhibits limited osteoinductive capability and insufficient mechanical strength for bone regeneration [23]. Meanwhile, 45S5 bioactive glass (BG) has attracted considerable attention because of its ability to release bioactive ions, including Si, Ca, and P species, which can stimulate osteogenic differentiation, promote vascularization, and buffer acidic degradation products [24], [25], [26]. The incorporation of BG into GelMA therefore provides an opportunity to introduce osteogenic and angiogenic functionality into a hydrogel matrix while retaining the favorable biological characteristics of GelMA [27].

Successful craniofacial bone regeneration also requires effective coordination between osteogenesis and angiogenesis [28], [29], [30]. Functional bone formation and vascular ingrowth are highly interdependent processes. Newly formed vasculature supplies oxygen, nutrients, and osteogenic signaling cues that support bone formation, while osteoblast-lineage cells release pro-angiogenic factors such as vascular endothelial growth factor (VEGF) to promote vascular ingrowth and maturation [31], [32], [33]. Previous studies have demonstrated that coordinated regulation of osteogenesis and angiogenesis significantly improves craniofacial bone repair. For example, de Carvalho et al. [34] developed 58S BG-containing MEW-PCL scaffolds that enhanced mineralized matrix formation and increased the expression of angiogenic markers, including CD31 and vWF, demonstrating their potential to support both osteogenic and angiogenic responses. Lv et al. [35] developed a temporally regulated silk fibroin hydrogel that sequentially promoted angiogenesis and osteogenesis for cranial bone regeneration. Despite these advances, achieving robust structural support while maintaining sustained regenerative bioactivity and coordinated osteogenic–angiogenic responses remain challenging for hydrogel-based scaffolds.

In this study, we developed a structurally optimized bioresorbable composite hydrogel to address the inherent trade-off between mechanical robustness and biological functionality in conventional hydrogels (Scheme 1). By integrating highly ordered MEW-fabricated poly(lactic-co-glycolic acid) (PLGA) scaffolds with a GelMA matrix incorporated with 45S5 BG, the composite hydrogels provide primary mechanical reinforcement through PLGA architecture while the BG-GelMA phase provides a bioactive microenvironment. This functional partitioning provides a practical means of partially decoupling mechanical reinforcement from hydrogel crosslinking. The resulting hydrogel was designed to support coordinated osteogenesis and angiogenesis for vascularized bone regeneration. The physicochemical properties, degradation behavior, mechanical performance, and *in vitro* osteogenic and angiogenic potentials were systematically characterized. Furthermore, transcriptome sequencing was employed to elucidate the underlying molecular mechanisms, and a rat critical-sized calvarial defect model was adopted to validate the *in vivo* bone repair efficacy of the optimized hydrogel.

**Scheme 1:**
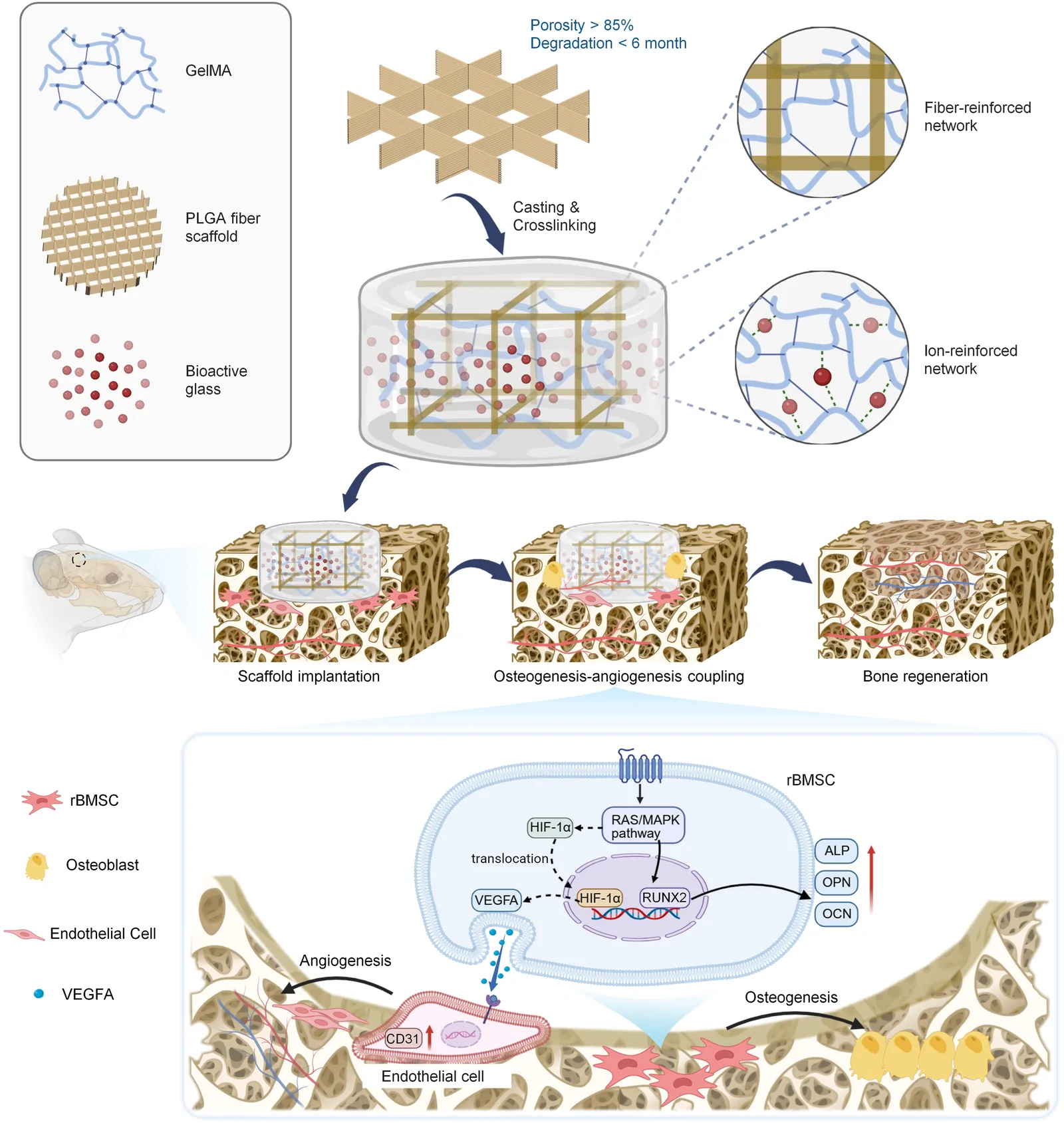
Schematic illustration of the synergistically reinforced bioresorbable hydrogel, integrating an architected PLGA microfibrous scaffold with a 45S5 BG-reinforced GelMA matrix to provide a strategy for partial mechanical–biological decoupling and osteogenic–angiogenic coupling for craniofacial bone regeneration. Created in BioRender. Xu, T. (2026) https://BioRender.com/okomc92.

## 2 Experimental section

### 2.1 Fabrication of composite hydrogel

#### 2.1.1 Materials

Medical-grade poly(lactic-co-glycolic acid) (PLGA, lactide-to-glycolide molar ratio = 78.6:21.4, *M*_w_ = 130,000 g/mol, PDI = 1.60; Batch No. TBZ3P182) was purchased from BMG (Kyoto, Japan). Gelatin methacryloyl (GelMA, EFL-GM-60) and photoinitiator lithium phenyl-2,4,6-trimethylbenzoylphosphinate (LAP) were purchased from EFL-Tech Co., Ltd. (Suzhou, China). 45S5 bioactive glass (BG), with a nominal composition of 45 wt% SiO_2_, 24.5 wt% Na_2_O, 24.5 wt% CaO, and 6 wt% P_2_O_5_, and particle sizes ranging from 20 to 50 μm, was purchased from Aladdin (B139900, Shanghai, China). Chloroform with a purity of 99% (CAS number: 67-66-3) was purchased from Nacalai Tesque, Inc. (Kyoto, Japan).

#### 2.1.2 Melt electrowriting (MEW) of PLGA scaffolds

Prior to printing, PLGA pellets underwent a two-stage drying process in a vacuum oven: heating at 80 °C for 2 h and then at 110 °C for an additional 12 h. The MEW device (MEW-TPS-260, Moxy Co., Ltd., Kyoto, Japan) was used for scaffold fabrication, and further specifications regarding this device can be found in our earlier publication [36]. The printing parameters included a melting temperature of 170 °C, a 27 G nozzle, a nitrogen pressure of 0.12 MPa, a collector temperature of 75 °C, a nozzle-to-collector distance of 2.5 mm, a voltage of 4.0 kV, and a printing speed of 7 mm s^−1^, resulting in a fiber diameter of 18 ± 1 μm. All printing experiments were conducted under conditions with a temperature of 23–25 °C and relative humidity of 30–35%.

#### 2.1.3 Hydrophilic treatment of PLGA scaffolds

The scaffolds were treated with NaOH solution (0.1 M) for 4.5 h to enhance their hydrophilicity. The treated scaffolds were then rinsed with deionized water to remove residual alkali, followed by a drying process at room temperature.

#### 2.1.4 Fabrication of PLGA/GelMA@BG composite hydrogels

A 5% (w/v) GelMA solution was prepared by blending GelMA into a phosphate buffered saline (PBS) solvent containing 0.25% (w/v) of the LAP, followed by heating at 65 °C for 25 min without light exposure. BG particles were then added into the GelMA solution at concentrations of 1, 5, 10, and 20 wt%, followed by stirring at 50 °C until a homogeneous suspension was obtained. The PLGA scaffolds were fully immersed in the GelMA@BG suspension and subsequently exposed to ultraviolet (UV) light for 10 s, forming PLGA/GelMA@BG (P/G@BG) composite hydrogels.

### 2.2 Characterization of composite hydrogel

#### 2.2.1 Swelling behavior

Swelling ratio was used to evaluate the swelling behavior of the hydrogels in phosphate-buffered saline (PBS, pH 7.4) at 37 °C, which was calculated based on the following equation:

(1) $$Swelling\;ratio=\frac{w_{t}-w_{0}}{w_{0}}\times 100\%$$

where *w*_0_ is the initial weight of the hydrogel, and *w*_t_ is the weight after treatment. Each sample was measured three times.

#### 2.2.2 Cyclic compression testing

Cyclic compression was conducted using a mechanical testing machine (STA-1150, Orientec Co. Ltd., Tokyo, Japan). The test was conducted for 10 repetitions at a loading speed of 5 mm min^−1^, wherein the strain was limited to 50% of the original sample thickness.

#### 2.2.3 Dynamic mechanical analysis (DMA)

DMA was characterized using a viscoelastic analyzer (Rheogel-E4000, UBM Co. Ltd., Kyoto, Japan) at 37 °C. The frequency sweeps started from 1 to 1000 Hz. For the tensile mode, composite specimens prepared by combining GelMA with PLGA scaffolds (25 mm in length and 5 mm in width) were tested with an effective gauge length of 15 mm under a minimum static load of 25 g. For the compression mode, cylindrical specimens (12 mm in diameter and 3 mm in thickness) were tested.

#### 2.2.4 Degradation behavior

The P/G@1BG composite hydrogels were immersed in PBS (pH 7.4) at 37 °C. The weight loss was calculated as follows:

(2) $$Weight\;loss=\frac{w_{0}-w_{t}}{w_{0}}\times 100\%$$

where *w*_0_ is the initial weight, *w*_t_ is the weight after degradation.

#### 2.2.5 Ion release from P/G@BG hydrogels

The composite hydrogels were immersed in deionized water at a ratio of 0.1 g/mL and maintained in a thermostatic shaker at 37 °C. The extracts were harvested and replaced with an equal volume of fresh deionized water at intervals of 1, 3, 5, 7, 14, and 21 days. The concentrations of Si, Ca, and P ions in the extracts were measured by inductively coupled plasma mass spectrometry (ICP-MS, PerkinElmer, USA).

#### 2.2.6 Alkaline hydrolysis

All polymer scaffolds were subjected to alkaline hydrolysis in NaOH (0.1 M) solution at 37 °C. The samples were harvested, thoroughly rinsed with deionized water, and subsequently dried for further evaluations at predetermined time points.

#### 2.2.7 Morphological observation

The PLGA scaffolds were sputter-coated with gold using a sputter coater (IB-2, Eiko Engineering Co., Ltd., Yamazaki, Japan) at 7 mA for 5 min for scanning electron microscope observation (SEM, Hitachi High-Tech Science Co., Japan) at an acceleration voltage of 10 kV. The hydrogels were dehydrated in a freeze-dryer (Alpha 2-4 LSCplus, Martin Christ Gefriertrocknungsanlagen GmbH, Germany) and were then coated with Au using a sputter coater (Quorum T150, Quorum Technologies, UK) for field-emission scanning electron microscope observation (FE-SEM, Nova Nano 450, FEI, USA) at an accelerating voltage of 5 kV. Element distributions on the hydrogel surface were analyzed by energy-dispersive X-ray spectroscopy (EDS) mode at an acceleration voltage of 10 kV.

#### 2.2.8 Water contact angle

A contact angle goniometer (Phoenix 300, S.E.O, Korea) was used to measure the water contact angle. For parallel comparison, a 3 μL droplet of deionized water was dropped on the sample surface.

#### 2.2.9 Gel permeation chromatography (GPC) analysis

The weight average molecular weights (*M*_w_) of PLGA and poly(ε-caprolactone) (PCL) were measured using gel permeation chromatography (GPC, Shimadzu Co., Ltd., Kyoto, Japan), and details were introduced in our previous study [37].

### 2.3 Biocompatibility evaluation

#### 2.3.1 Evaluation of in vitro cytocompatibility and hemocompatibility

Hydrogel extracts were prepared according to ISO 10993-12 by immersing hydrogels in α-MEM (Gibco, USA) at a ratio of 0.1 g mL^−1^ and incubating at 37 °C for 24 h with gentle agitation. The extracts were subsequently sterilized using a 0.22 μm membrane filter. For cytocompatibility evaluation, rat bone marrow mesenchymal stem cells (rBMSCs) were seeded in 24-well plates at a density of 6 × 10^4^ cells per well and cultured overnight. The culture medium was then replaced with complete medium containing 100% hydrogel extract. On days 1, 3, and 5, Cell Counting Kit-8 (CCK-8) assay (GK10001, GLPBIO, USA) was performed. Absorbance was quantified at 450 nm with a microplate reader (Thermo Variscan, Thermo Fisher Scientific, Waltham, MA, USA), followed by calculation of cell viability using the equation below:

(3) $$Viability=\frac{A_{s}-A_{b}}{A_{c}-A_{b}}\times 100\%$$

where *A*_*s*_, *A*_*c*_, and *A*_*b*_ correspond to the absorbance of the sample, control, and blank groups, respectively. Live/dead staining was conducted with a Live/Dead Cell Staining Kit (C2015S, Beyotime, China) according to the manufacturer's instructions, and images were acquired using an inverted fluorescence microscope (IX73, Olympus Corporation, Tokyo, Japan).

Hemolysis was evaluated according to ISO 10993-4 using rat venous blood diluted with normal saline at a ratio of 1:1.25 (v/v). Hydrogel extracts (1 mL) were mixed with 50 μL of diluted blood and incubated at 37 °C for 60 min, with deionized water and normal saline serving as positive and negative controls. Following centrifugation at 3000 rpm for 20 min, the absorbance of the collected supernatant was determined at 540 nm. The hemolysis rate was calculated using the following equation:

(4) $$Hemolysis\;rate=\frac{W_{1}-W_{2}}{W_{0}-W_{2}}\times 100\%$$

where *W*_*1*_, *W*_*0*_, and *W*_*2*_ represent the absorbance of the experimental, positive control, and negative control groups, respectively.

#### 2.3.2 Cell morphology observation

rBMSCs were seeded on different hydrogels at a density of 6 × 10^4^ cells per well. After 5 days of culture, cells were rinsed with PBS and subsequently fixed with 4% paraformaldehyde for 20 min. The cytoskeletal F-actin and cell nuclei were fluorescently labeled using Phalloidin-iFluor 488 (PF00001, Proteintech, USA) and DAPI (P0131, Beyotime, China), respectively. Cellular morphology was observed using a confocal laser scanning microscope (STELLARIS 5, Leica Microsystems, Mannheim, Germany), and cell spreading area was quantified using ImageJ software (National Institutes of Health, USA).

### 2.4 *In vitro* osteogenic differentiation evaluation

#### 2.4.1 Alkaline phosphatase (ALP) staining and quantification

To evaluate the *in vitro* osteogenic bioactivity of P/G@BG hydrogels, rBMSCs were either cultured with hydrogel extracts or directly seeded onto the hydrogel substrates. ALP staining was performed using a commercial kit (C3206, Beyotime, China) after 7 and 14 days of incubation. For quantification, cells were lysed, and ALP activity and total protein content were measured using ALP and bicinchoninic acid (BCA) protein assay kits (P0321S and P0010S, Beyotime, China), respectively. ALP activity was normalized to total protein content.

#### 2.4.2 Alizarin Red S (ARS) staining and quantification

Matrix mineralization was assessed by ARS staining on day 21 using the same two culture methods described in Section 2.4.1. Cells were fixed with 4% paraformaldehyde and stained with ARS solution (G1452, Solarbio, China) according to the manufacturer's instructions. Following ARS staining, calcium deposits were solubilized with 10% cetylpyridinium chloride (C129534, Aladdin, China), and the absorbance was quantified at 562 nm using a microplate reader.

#### 2.4.3 qRT-PCR

Total RNA was isolated using the TRIzol® Plus RNA Purification Kit (12183-555, Thermo Fisher Scientific, USA) and quantified with a NanoDrop spectrophotometer. Subsequently, 1 μg of RNA was reverse transcribed into cDNA using the SuperScript™ III First-Strand Synthesis SuperMix (11752-050, Thermo Fisher Scientific, USA). qRT-PCR analysis was performed on a CFX384 Real-Time PCR System (CFX384, Bio-Rad, USA) with Power SYBR® Green PCR Master Mix (4367659, Applied Biosystems, USA) using specific primer sets for GAPDH, ALP, Runx2, OCN, OPN, BMP-2, COL1A1, VEGFA, and HIF-1α (Table 1). Relative gene expression levels were determined using the 2^−ΔΔCt^ method, with GAPDH serving as the reference gene.

**Table 1:** Primer sequences used for qRT-PCR analysis.

Gene	Forward Primer	Reverse Primer
GAPDH	GAAGGTCGGTGTGAACGGATTTG	CATGTAGACCATGTAGTTGAGGTCA
ALP	CCTAGACACAAGCACTCCCACTA	GGTCAGTCAGGTTGTTCCGATTC
Runx2	CATCCTTCCCTCCGAGACCCTAA	CCCAACATGGCTGCTCCCTTC
OPN	CAAGGTCATCCCAGTTGCCCAG	GCTTTGGAACTCGCCTGACTGTC
BMP2	ACAGCTGGTCTCAGGTAAGACCAC	CCATGGCAGTAAAAGGCATGATAGCCT
OCN	CTCACTCTGCTGGCCCTGAC	CACCTTACTGCCCTCCTGCTTG
COL1A1	TGCGATGGCGTGCTATGC	GACTCCTATGACTTCTGCGTCT
VEGFA	GTCACCACCACACCACCATCGT	CTCCTCTCCCTTCATGTCAGGCT
HIF-1α	CCCAGAGTCACTGGGACTT	CTGTTAGGCTCAGGTGAGCTT

#### 2.4.4 Immunofluorescence staining

After 14 days of osteogenic induction, rBMSCs were fixed with 4% paraformaldehyde, permeabilized with enhanced immunostaining permeabilization buffer (P0097, Beyotime, China), and blocked with 5% bovine serum albumin (BSA) (SW3015, Solarbio, China). Cells were incubated overnight at 4 °C with primary antibodies against Runx2 (ab192256, 1:1000, Abcam, UK) and OCN (NBP3-16081, 1:100, Novus, USA), followed by incubation with Alexa Fluor® 594-conjugated secondary antibodies (ab150080, 1:200, Abcam, UK). F-actin and nuclei were counterstained with Phalloidin-iFluor 488 and DAPI, respectively. Confocal laser scanning microscopy was used to obtain fluorescence images.

### 2.5 Migration and vascularization ability of HUVECs

#### 2.5.1 Wound healing assay

To evaluate the paracrine pro-angiogenic effect of hydrogel-stimulated rBMSCs, cells were cultured on different hydrogels in FBS-free α-MEM for 3 days. The supernatants were harvested, centrifuged, filtered through a 0.22 μm membrane, mixed with DMEM at a 1:1 ratio, and supplemented with 2% FBS to prepare conditioned medium (CM) from each group. Human umbilical vein endothelial cells (HUVECs) were seeded in 12-well plates at 3 × 10^5^ cells per well and grown to confluence. A uniform scratch was made using a sterile pipette tip, washed with PBS. HUVECs were then incubated with different CM for 24 h, and scratch closure was quantified using ImageJ software.

#### 2.5.2 Tube formation assay

Prior to use, Matrigel was thawed on ice and evenly dispensed into pre-cooled 96-well plates. After gelation at 37 °C, HUVECs were resuspended in CM derived from rBMSCs cultured on different hydrogels, and seeded onto the Matrigel matrix at 1 × 10^4^ cells per well. Cells cultured in DMEM supplemented with 2% FBS served as the control. Following 6 h of incubation, tube formation was observed under a microscope, and relevant parameters were analyzed using ImageJ software.

### 2.6 *In vivo* animal study

#### 2.6.1 Calvarial defect model establishment

All animal procedures were approved by the Animal Ethics Committee of the First Affiliated Hospital, Zhejiang University School of Medicine (Approval No.20241725). Eighteen 12-week-old Sprague-Dawley rats (350 ± 50g) were housed under specific pathogen-free conditions. Under sterile conditions and isoflurane anesthesia, a midline scalp incision was made, and bilateral full-thickness calvarial defects (5 mm in diameter) were established using a dental trephine. The bilateral calvarial defects in each rat were set as paired experimental sites and randomly assigned into three groups: blank control, P/G and P/G@1BG. Cranial specimens were harvested at 8 and 12 weeks post-implantation, and major visceral organs were collected at 8 weeks for H&E staining. All specimens were fixed in 4% paraformaldehyde for subsequent histological processing.

#### 2.6.2 Micro-CT analysis

The harvested bone specimens were scanned using a micro-CT system (Skyscan, Bruker, Kontich, Belgium). Three-dimensional reconstruction models were generated based on sagittal, axial, and coronal sections. Quantitative bone regeneration parameters including bone volume to total volume ratio (BV/TV), bone mineral density (BMD), trabecular number (Tb.N) and trabecular separation (Tb.Sp), were analyzed using the manufacturer's software.

#### 2.6.3 Histological, immunohistochemical, and immunofluorescence analysis

Following complete ethylenediaminetetraacetic acid (EDTA) decalcification, bone specimens were dehydrated, paraffin-embedded, and serially sectioned. H&E and Masson's trichrome staining (Sigma-Aldrich, USA) were performed to characterize the microstructure of newly regenerated bone tissue. For immunohistochemical detection, sections were incubated with primary antibodies against Runx2 (ab236639, 1:100, Abcam, UK) and CD31 (ab281583, 1:1000, Abcam, UK), followed by corresponding secondary antibodies incubation. Subsequent DAB chromogenic development and hematoxylin counterstaining were conducted for histological observation. Additionally, double immunofluorescence staining of Runx2 and CD31 was carried out to evaluate the spatial correlation between osteogenesis and angiogenesis, and the corresponding fluorescence intensity was quantified using ImageJ software.

### 2.7 Transcriptome sequencing and bioinformatic analysis

Transcriptome sequencing was performed to analyze the mRNA expression profiles of rBMSCs cultured on control group and P/G@1BG hydrogels. Total RNA was extracted using TRIzol reagent, and its concentration and purity were assessed using a NanoDrop spectrophotometer. Qualified RNA samples were subjected to mRNA library construction and high-throughput sequencing on the Illumina platform (Beijing Zhongxing Biomedi Technology Co., Ltd., China). Differentially expressed genes (DEGs) were identified using DESeq2 with the threshold of adjusted *p* < 0.05 and |fold change| > 1.2. Gene Ontology (GO) and Kyoto Encyclopedia of Genes and Genomes (KEGG) enrichment analyses of upregulated DEGs were performed using the clusterProfiler R package. Terms and pathways with adjusted *p* < 0.05 were considered significantly enriched.

### 2.8 Statistical analysis

Each experiment included at least three independent biological replicates. Data are reported as the mean with standard deviation. Statistical calculations were conducted in SPSS 22.0. Differences among multiple groups were examined by one-way ANOVA with Tukey's multiple-comparison procedure, and statistical significance was assigned at *p* < 0.05.

## 3 Results and discussion

### 3.1 Structure-directed mechanical reinforcement

The highly ordered poly(lactic-co-glycolic acid) (PLGA) microfibrous scaffolds were fabricated by melt electrowriting (MEW), in which a molten polymer jet was continuously stretched under an electrostatic field to produce precisely deposited microfibers (Fig. 1A). Owing to the high printing fidelity of MEW, the resulting scaffolds possessed a porosity exceeding 85% while maintaining well-defined interconnected architectures with controllable pore geometries. The ultralight nature of the PLGA scaffold was demonstrated by placing it on a fragile dandelion without causing visible deformation (Fig. 1B).

**Fig. 1:**
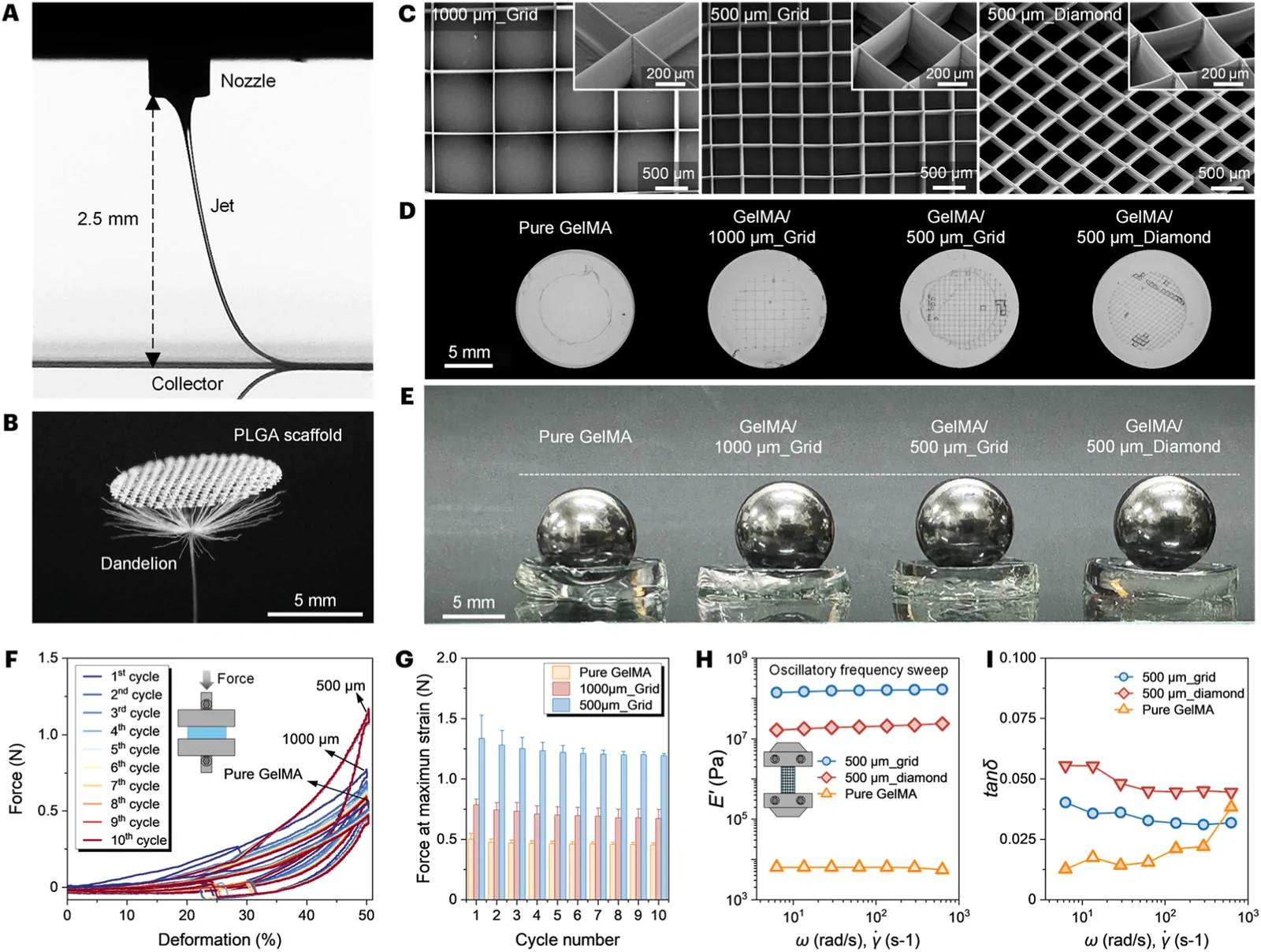
Structure-directed mechanical reinforcement enabled by architected PLGA microfibrous scaffolds. (A) Representative photograph of the melt electrowriting process. (B) Photograph showing an ultralight PLGA scaffold placed on a dandelion. (C) SEM images of MEW-printed PLGA scaffolds with different lattice architectures. (D) Representative photograph of the PLGA/GelMA composite hydrogels after GelMA infiltration and photocrosslinking. (E) Qualitative comparison of the load-bearing capability of the composite hydrogels using a steel ball. (F) Cyclic compression force–deformation curves over ten loading cycles and the corresponding (G) maximum compression force. DMA results in tensile mode showing (H) storage modulus (*E′*) and (I) loss factor (tan*δ*) of pure GelMA and composite hydrogels with different lattice architectures.

To investigate the role of architecture in regulating the mechanical behavior of the composite hydrogel, three representative lattice geometries, including a 1000 μm square grid, a 500 μm square grid, and a 500 μm diamond lattice, were fabricated (Fig. 1C). All scaffolds exhibited highly uniform fiber diameters, accurate layer stacking, and complete interfiber fusion. Detailed morphologies obtained via optical microscopy and scanning electron microscopy (SEM) are shown in Fig. S1. Before hybridization with the hydrogel, the PLGA scaffolds underwent surface modification to enhance their hydrophilicity, achieving complete water wetting as shown by contact angle measurements (Fig. S2). After infiltration with the gelatin methacryloyl (GelMA) precursor solution and subsequent photocrosslinking, the hydrogel completely filled the interconnected pores of the PLGA scaffold, forming an integrated composite without interfacial separation (Fig. 1D). The resulting composites exhibited dramatically improved load-bearing capability compared with pure GelMA, as qualitatively demonstrated by supporting a steel ball without noticeable deformation (Fig. 1E). The load-bearing capability was further quantified by cyclic compression tests. As shown in Fig. 1F and G, decreasing the fiber spacing from 1000 to 500 μm significantly increased the maximum compressive force throughout ten loading cycles, as increasing the fiber density effectively generated more continuous load-transfer pathways throughout the composite.

To further elucidate the influence of architectural geometry, dynamic mechanical analysis (DMA) was performed in tensile mode using the two scaffolds with identical fiber density but different lattice structures. Remarkably, although both structures exhibited comparable compressive resistance, the square lattice displayed a storage modulus (*E′*) exceeding 100 MPa, which was approximately one order of magnitude higher than that of the diamond lattice (Fig. 1H). Meanwhile, the storage modulus of the composite hydrogels was several orders of magnitude higher than that of pure GelMA (∼6 kPa), demonstrating the strong reinforcing effect of the PLGA scaffold. DMA in tensile mode revealed distinct viscoelastic behaviors. The fiber-reinforced hydrogels retained predominantly elastic behavior with a low loss factor (tan*δ* ∼ 0.05), whereas pure GelMA also exhibited predominantly elastic behavior with a low loss factor (tan*δ* ∼ 0.01–0.04) (Fig. 1I), indicating that the incorporation of the fiber architecture substantially altered the mechanical response of the hydrogel under tensile loading.

Therefore, the mechanical performance of the hydrogel could be effectively regulated by simply altering the three-dimensional architecture of the PLGA scaffold without changing the GelMA composition and polymer chemistry. By adjusting fiber spacing or lattice geometry, compressive strength, tensile stiffness, and viscoelastic behavior were continuously tuned while maintaining an identical hydrogel formulation. Notably, the PLGA scaffold accounted for only 1 wt% of the entire construct, yet substantially enhanced the mechanical response of the hydrogel. This finding highlights the role of the architected microfiber network as a structure-directed mechanical reinforcement, shifting the contribution of bulk mechanics from the hydrogel matrix alone toward the combined effects of molecular crosslinking and spatial architecture. Such architecture-guided reinforcement establishes the foundation for the subsequent mechanical–biological decoupling strategy, in which mechanical robustness is primarily provided by the PLGA scaffold while the BG-GelMA matrix is retained as the cell-compatible and bioactive phase.

### 3.2 Ion-mediated network reinforcement

Following the establishment of the structure-directed PLGA/GelMA (P/G) hydrogel, the incorporation of 45S5 bioactive glass (BG) can further reinforce the hydrogel. SEM observations revealed an interconnected porous architecture without noticeable interfacial separation between the PLGA fibers and GelMA hydrogel (Fig. 2A and Fig. S3), indicating effective integration of the architected PLGA scaffold with the surrounding hydrogel. Meanwhile, Ca elemental mapping further confirmed the homogeneous distribution of BG particles throughout the GelMA hydrogel, even at a loading of 20 wt%, without obvious particle aggregation, providing a prerequisite for uniform mechanical reinforcement and sustained ion release.

**Fig. 2:**
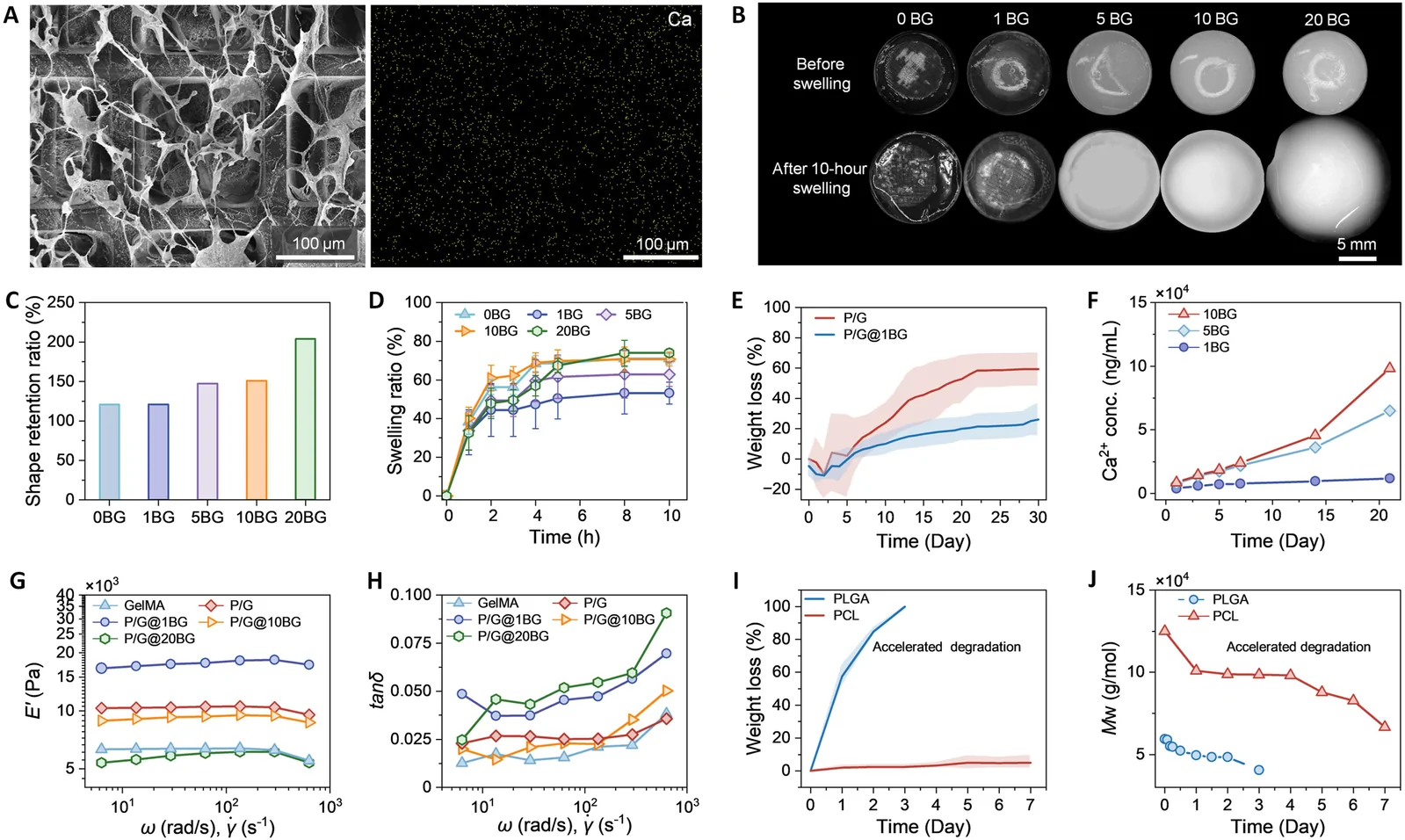
Ion-mediated network reinforcement and degradation of the composite hydrogels. (A) SEM image of the freeze-dried P/G@20BG hydrogel and the corresponding Ca elemental mapping. (B) Representative photographs of hydrogels containing different BG contents before and after immersion in PBS for 10 h, together with the corresponding (C) shape retention ratio and (D) swelling ratio. (E) Weight loss profiles of P/G and P/G@1BG hydrogels in PBS. (F) Cumulative Ca^2+^ release from hydrogels with different BG contents. DMA showing (G) storage modulus (*E′*) and (H) loss factor (tan*δ*). Accelerated alkaline degradation of PLGA and PCL was evaluated by the changes in (I) weight loss and (J) molecular weight (*M*_w_).

The structural stability of the hydrogels strongly depended on the BG content. After immersion in PBS for 10 h, the hydrogels containing 1–10 wt% BG largely maintained their original geometries, whereas the P/G@20BG hydrogel exhibited pronounced structural collapse (Fig. 2B). This trend was quantitatively confirmed by the shape retention ratio (Fig. 2C). Although the P/G hydrogel showed limited dimensional change, its swelling ratio was relatively high (Fig. 2D). Among all groups, P/G@1BG showed the lowest swelling while maintaining excellent structural integrity, suggesting that a moderate amount of BG effectively restricted excessive network expansion during hydration. Consistently, the incorporation of 1 wt% BG markedly delayed hydrogel degradation, reducing the weight loss after 28 days from approximately 60% for P/G to about 25% for P/G@1BG (Fig. 2E). The reduced degradation rate is attributed to the suppression of excessive swelling, which limits water penetration into the hydrogel network and consequently reduces hydrolytic degradation.

Beyond its effect on swelling and degradation, BG also served as a source of gradually released inorganic ions. As shown in Fig. 2F, Ca^2+^ release exhibited a continuous and BG-content-dependent profile over 21 days, with similar behavior observed for P and Si ions (Fig. S4). The sustained release was likely associated with the gradual degradation of the GelMA matrix and dissolution of the embedded BG particles, which progressively exposed the inorganic phase to the surrounding medium. The released Ca^2+^ may further interact with functional groups in the GelMA network. Similar ion–polymer interactions have been inferred from FTIR analyses of gelatin/BG composites, in which spectral changes were associated with interactions between carboxyl groups and BG-derived Ca^2+^ [38]. Ca^2+^–carboxyl interactions have also been linked to polymer-chain association and changes in swelling behavior in gelatin-based networks [39]. These interactions may therefore contribute to the reduced network expansion and improved dimensional stability observed at moderate BG loading.

DMA results further supported this reinforcement mechanism. As shown in Fig. 2G, the addition of 1 wt% BG further increased *E′* compared to the fiber-reinforced GelMA. However, further increasing the BG content resulted in a gradual reduction in storage modulus. Excessive BG particles disrupted the continuity of the GelMA network and reduced the effective polymer fraction available for photocrosslinking, thereby weakening the integrity of the hydrogel despite the increased number of particle-polymer interactions. Consequently, an optimal BG concentration was identified, where network reinforcement arising from competitive molecular interactions was maximized without compromising network continuity. The loss factor (tan*δ*) remained low for all groups (Fig. 2H), indicating that the composite hydrogels preserved their predominantly elastic behavior despite BG incorporation. Therefore, moderate BG incorporation not only reinforced the hydrogel mechanically but also preserved its elasticity, which is essential for maintaining structural integrity during swelling and degradation.

To further demonstrate the advantage of using PLGA as the structural reinforcement phase, accelerated alkaline hydrolysis was performed to compare the degradation behaviors of PLGA and poly(*ε*-caprolactone) (PCL). PLGA degraded faster than PCL, exhibiting 60% weight loss after one day and completely losing its structural integrity within three days, whereas PCL showed less than 6% weight loss after seven days (Fig. 2I). The *M*_w_ of PLGA decreased from 59,300 to 40,400 g mol^−1^ within three days, and the *M*_w_ of PCL decreased from 125,000 to 66,700 g mol^−1^ within seven days (Fig. 2J). The progressive reduction in molecular weight was further supported by the corresponding Gel Permeation Chromatography (GPC) chromatograms, which exhibited a distinct shift of elution peaks toward longer retention times over the degradation period (Fig. S5). These results indicate the greater hydrolytic susceptibility of PLGA relative to PCL under accelerated conditions. Importantly, previous studies under physiological and *in vivo* conditions have also demonstrated progressive degradation of PLGA scaffolds over several weeks to months, accompanied by decreases in molecular weight, structural integrity, and mechanical properties [40], [41]. Together, these findings support the use of PLGA as a bioresorbable reinforcing phase whose structural contribution can progressively diminish during tissue regeneration.

### 3.3 *In vitro* cytocompatibility and hemocompatibility of composite hydrogel

Superior cytocompatibility serves as a fundamental prerequisite for the translational application of biomaterials, with a viability rate above 75% generally regarded as non-cytotoxic [42]. CCK-8 assays (Fig. 3A) demonstrated that rat bone marrow mesenchymal stem cells (rBMSCs) cultured on P/G, P/G@1BG, and P/G@5BG hydrogels maintained stable and high cell viability throughout the culture period. Notably, the P/G@1BG group exhibited enhanced proliferative activity, with significantly higher cell viability than the P/G group from day 3 onwards and the highest viability at day 5. This indicates that moderate BG incorporation effectively optimizes the bioactive microenvironment without inducing adverse cellular responses. In contrast, P/G@10BG and P/G@20BG hydrogels markedly suppressed cell proliferation, leading to a significant decline in viability at the later culture stage.

**Fig. 3:**
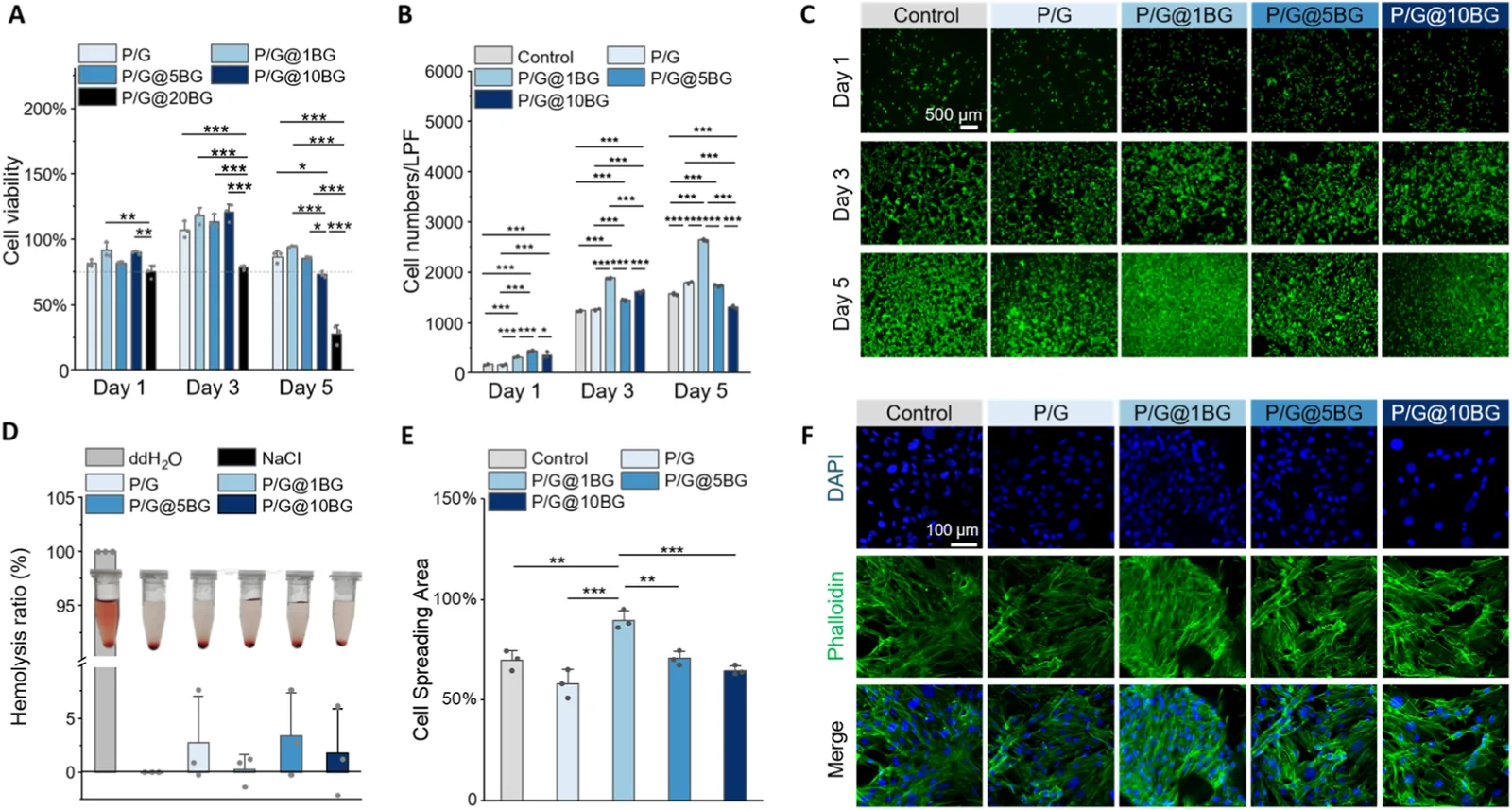
Biocompatibility and cell adhesion on P/G@BG composite hydrogels. (A) rBMSCs viability assessed by CCK-8 assay. (B) Quantitative analysis of cell numbers per low-power field (LPF) derived from live/dead staining. (C) Live/dead fluorescence staining of rBMSCs cultured with different hydrogel extracts (green: live cells, red: dead cells). (D) Hemolysis ratio of rat erythrocytes incubated with hydrogel extracts. (E) Semi-quantitative analysis of cell spreading area. (F) F-actin staining of rBMSCs on different hydrogels (green: F-actin; blue: nuclei). *p < 0.05, **p < 0.01, ***p < 0.001.

Live/dead fluorescent staining further validated the quantitative findings (Fig. 3B and C). The control, P/G, P/G@1BG, and P/G@5BG groups displayed abundant viable cells with negligible dead cell signals, suggesting favorable cellular compatibility. The P/G@1BG group supported the most uniform and dense cell distribution, further confirming its superior capacity to sustain rBMSCs expansion. Although the P/G@10BG group maintained relatively high cell viability at the initial stage, it exhibited reduced cell proliferation during prolonged culture, as evidenced by the lower viable cell density at day 5 compared with the P/G@1BG and P/G@5BG groups, consistent with the CCK-8 results. The reduced cell proliferation at high BG contents (10–20 wt%) may be associated with the increased particle loading, which can alter the local ionic and pH environment, while the 20–50 μm particles may additionally contribute to microscale surface heterogeneity [43], [44], [45].

The hemocompatibility of the hydrogels was further assessed to evaluate their biosafety. The hemolysis ratios of all P/G and P/G@BG hydrogels were significantly below the 5% threshold (Fig. 3D), comparable to the NaCl negative control. No noticeable erythrocyte lysis or macroscopic hemolysis was observed in any of the hydrogel groups. These results demonstrate that the composite hydrogels, particularly the P/G@1BG formulation, exhibit excellent cytocompatibility, thereby meeting the essential biosafety standards for subsequent *in vivo* applications.

### 3.4 rBMSCs adhesion and cytoskeletal organization on composite hydrogels

Cell adhesion and cytoskeletal organization are critical early events that determine subsequent cell behavior and fate decisions. Phalloidin/DAPI staining (Fig. 3F) revealed that rBMSCs cultured on P/G, P/G@1BG, and P/G@5BG hydrogels exhibited an elongated and well-spread morphology, with organized actin bundles and interconnected cellular networks on the hydrogel surfaces. Semi-quantitative analysis of cell spreading area (Fig. 3E) confirmed that the P/G@1BG group exhibited the most extensive spreading among all groups. In contrast, the P/G@10BG group exhibited reduced spreading relative to the lower BG-loading groups and was accompanied by reduced proliferation. Based on these results, the 1–5 wt% BG range was determined to be the optimal loading for achieving a balance between bioactivity and cellular safety.

The enhanced cell spreading and actin alignment on P/G@1BG hydrogels are attributed to the microarchitectural guidance provided by the MEW-printed PLGA scaffold rather than increased hydrogel crosslinking. The GelMA/BG matrix concurrently preserves a cytocompatible microenvironment through its hydrophilicity, cell-adhesive motifs, and sustained ion release. This structured cytoskeletal arrangement enhances osteogenic differentiation, as mechanical tension generated by aligned actin filaments promotes nuclear translocation of osteogenic transcription factors to regulate downstream target genes [46].

### 3.5 *In vitro* osteogenic differentiation of composite hydrogels

The osteogenic potential of the composite hydrogels was assessed *in vitro* to evaluate their capacity for bone regeneration. Alkaline phosphatase (ALP) staining showed that the incorporation of BG significantly enhanced early osteogenic differentiation of rBMSCs (Fig. 4A). The staining intensity increased markedly from day 7 to day 14 across all experimental groups (Fig. S6). Quantitative analysis demonstrated that the P/G@1BG group exhibited the highest ALP activity at day 7, approximately 1.6 times that of the blank control group (Fig. 4B). Similar tendencies were further confirmed in direct cell seeding experiments (Fig. S7). Alizarin Red S (ARS) staining performed on day 21 showed massive calcium nodule formation in both P/G@1BG and P/G@5BG groups (Fig. 4C), and consistent outcomes were observed in direct cell seeding assays (Fig. S8). Quantitative detection further demonstrated that the P/G@1BG group achieved the highest mineralization capacity (Fig. 4D).

**Fig. 4:**
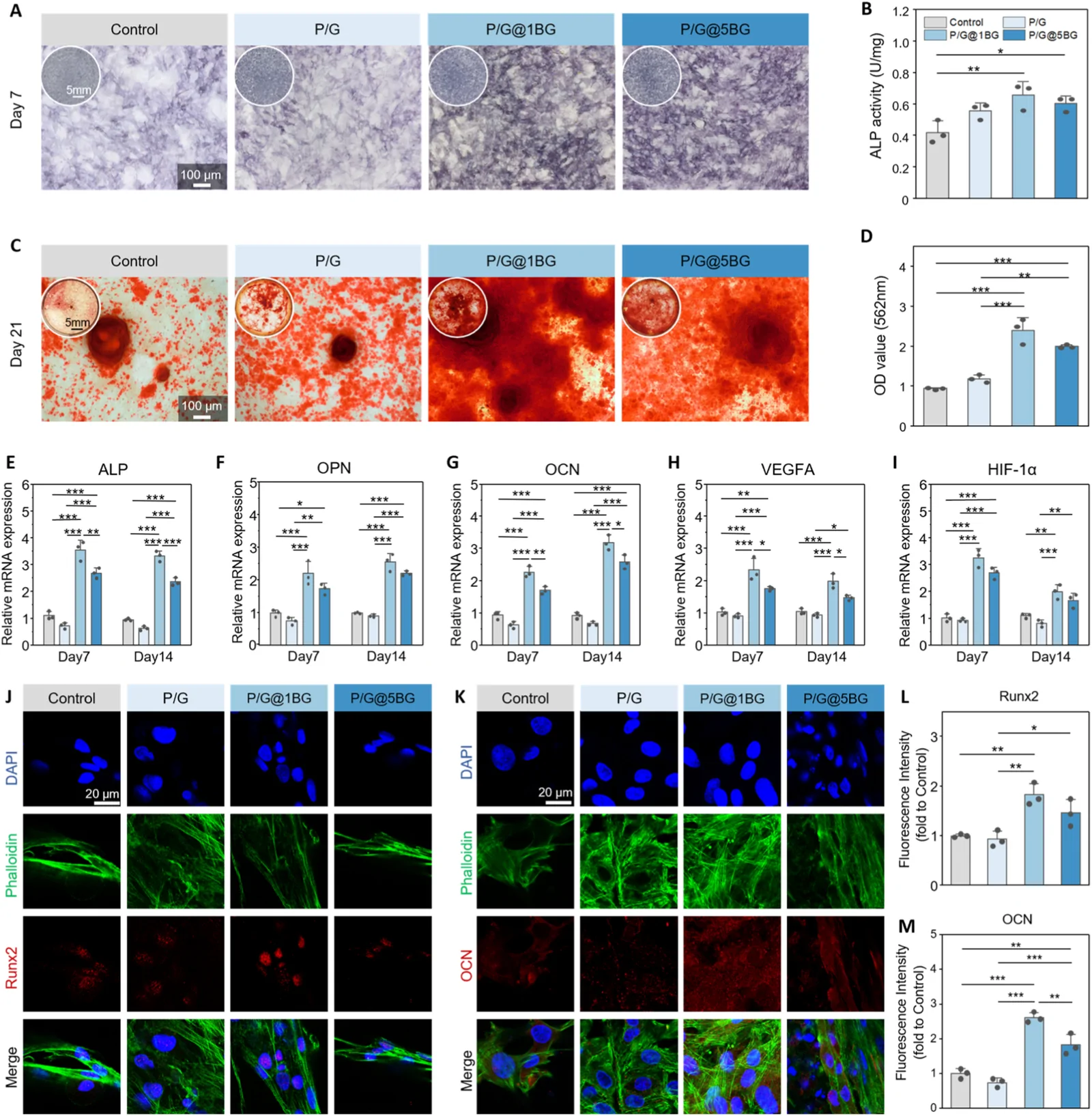
*In vitro* osteogenic differentiation of rBMSCs on P/G@BG composite hydrogels. (A) ALP staining at day 7. (B) Quantitative analysis of ALP activity. (C) ARS staining at day 21. (D) Quantitative analysis of ARS staining. (E–I) qRT-PCR analysis of osteogenic genes (ALP, OPN, and OCN) and angiogenesis-related genes (VEGFA and HIF-1α) at 7 and 14 days. (J, K) Immunofluorescence staining of Runx2 and OCN at day 14. (L, M) Semi-quantitative analysis of Runx2 and OCN fluorescence intensity. *p < 0.05, **p < 0.01, ***p < 0.001.

The expression profiles of key osteogenic genes, including ALP, BMP-2, OCN, OPN, Runx2, and COL1A1, were further analyzed by quantitative reverse transcription polymerase chain reaction (qRT-PCR) at days 7 and 14 (Fig. 4E–G and Fig. S9). All osteogenic genes were significantly upregulated in the P/G@1BG group at both time points. The early osteogenic marker ALP reached peak expression on day 7, whereas the mid-late-stage markers OPN and OCN remained highly expressed through day 14. This pattern indicates the complete progression of rBMSCs from osteogenic lineage commitment to terminal functional maturation.

Immunofluorescence staining of Runx2 and OCN further verified the above molecular results (Fig. 4J and K). Quantitative analysis showed that the relative expression levels of Runx2 and OCN in the P/G@1BG group were approximately 1.8-fold and 2.6-fold those of the blank control group, respectively (Fig. 4L and M). Subcellular localization analysis demonstrated that Runx2 was predominantly localized within the nucleus, aligning with its function in regulating osteogenic transcriptional programs. Conversely, OCN was extensively expressed in the cytoplasm, confirming the successful maturation of rBMSCs into functional osteoblasts. Collectively, these *in vitro* results suggest that P/G@BG hydrogels establish a conducive bioactive microenvironment that promotes osteogenic differentiation and maturation of rBMSCs.

The superior osteogenic capacity of the composite hydrogels arises from the synergistic contribution of each constituent. Specifically, the MEW-fabricated PLGA scaffold provides biomimetic topography that regulates cellular mechanotransduction behavior [47], [48]. The GelMA matrix imparts favorable hydrophilicity and abundant arginine–glycine–aspartic acid (RGD) cell-adhesive motifs to the composite system, supporting stable cell adhesion and sustained cell viability [37]. Moreover, 45S5 BG incorporated within the matrix sustains the release of Si, Ca, and P species, which activate osteogenic signaling and promote mineralized interface formation. Structurally, overall mechanical performance is governed by the PLGA microfiber network rather than by hydrogel crosslinking, thereby preserving the bioactivity of the GelMA and BG components. This architectural design provides a strategy for partial mechanical–biological decoupling, wherein structural reinforcement maintains the biological functionality of the hydrogel matrix.

### 3.6 *In vitro* angiogenic activation via rBMSCs-derived paracrine signals

Vascular ingrowth is a critical rate-limiting process in functional bone regeneration, sustaining nutrient delivery, cell recruitment, and metabolic waste clearance [49]. This challenge is particularly pronounced in critical-sized craniofacial bone defects due to the inherently inadequate local vascular network. Therefore, synergistic modulation of osteogenesis and angiogenesis has become a core design principle for high-performance craniofacial bone repair hydrogels [50]. qRT-PCR results demonstrated significant upregulation of VEGFA and HIF-1α in rBMSCs at day 7, especially in the P/G@1BG group, indicating pro-angiogenic paracrine activation (Fig. 4H and I). To verify this effect, conditioned medium (CM) from rBMSCs cultured on different hydrogels was collected and applied to human umbilical vein endothelial cells (HUVECs) for wound healing and tube formation assays.

As shown in Fig. 5A and B, the wound closure rate reached approximately 75.6% after 24 h incubation with P/G@1BG-derived CM, significantly higher than those of the P/G@5BG-CM group (70.7%), P/G-CM group (45.4%) and blank control group (50.0%). Identical trends were observed in tube formation assays (Fig. 5C–5E), where HUVECs stimulated by P/G@1BG-CM formed the most integrated capillary-like networks, accompanied by increased total tube length and branching junction numbers. These findings confirm that P/G@1BG hydrogels can effectively stimulate rBMSCs to secrete soluble pro-angiogenic paracrine factors.

**Fig. 5:**
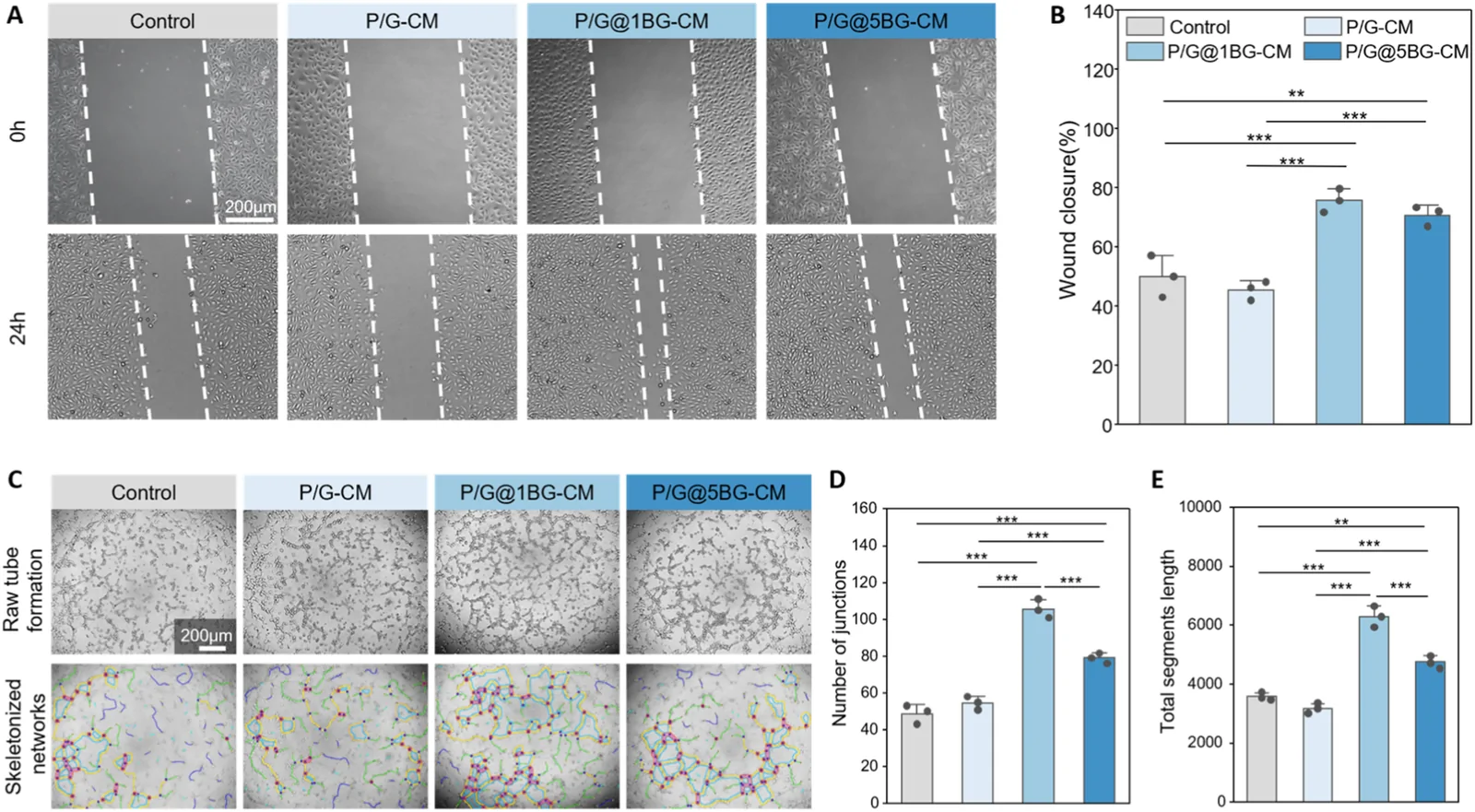
*In vitro* angiogenic potential evaluation. (A) HUVECs wound healing assay images at 0 and 24 h. (B) Quantitative analysis of wound closure rate at 24 h. (C) Representative HUVECs tube formation assay images after 6 h of culture; red dots mark junctions (three-capillary like structure intersections), yellow lines denote segments (junction connections), and closed circles represent meshes. (D) Quantitative analysis of the number of junctions. (E) Quantitative analysis of total tube length. *p < 0.05, **p < 0.01, ***p < 0.001.

Combined with the elevated transcription of HIF-1α and VEGFA in hydrogel-cultured rBMSCs, these results further demonstrate that the enhanced angiogenic capacity is primarily mediated by BMSC-released VEGFA, which activates the biological functions of endothelial cells. This paracrine cellular interaction establishes an inherent regulatory link between the osteogenic differentiation of rBMSCs and endothelial cell activation, serving as the fundamental molecular basis for osteogenesis-angiogenesis coupling [32], [51]. Such a favorable regulatory mechanism possesses great application potential for repairing large craniofacial bone defects with inadequate endogenous vascularization [52].

### 3.7 *In vivo* Osteogenic–angiogenic coupling in a rat calvarial defect model

Based on the superior *in vitro* bioactivities, P/G@1BG showed superior overall performance in cell viability, osteogenic differentiation, and pro-angiogenic activity. Therefore, the optimized P/G@1BG hydrogel was implanted into rat calvarial defects to evaluate its *in vivo* bone regenerative performance. Prior to functional evaluation, *in vivo* biosafety was assessed via H&E staining of major visceral organs. No obvious pathological lesions, inflammatory infiltration or tissue necrosis were observed in the P/G@1BG group, and the overall organ morphology was consistent with that of the control group, confirming its favorable *in vivo* biocompatibility (Fig. S10).

Micro-CT three-dimensional reconstruction and coronal section images showed that new bone grew from defect margins toward the central region in all groups (Fig. 6A). Composite hydrogel implantation markedly accelerated endogenous bone regeneration relative to the blank control. At 12 weeks post-implantation, the bone coverage rate reached approximately 60% in the P/G@1BG group, while the P/G and blank control groups only reached 40% and 25%, respectively. Quantitative analysis of key micro-CT parameters including bone volume to total volume ratio (BV/TV), bone mineral density (BMD), trabecular number (Tb.N) and trabecular separation (Tb.Sp), further confirmed the superior *in vivo* osteogenic capacity of the composite hydrogels (Fig. 6C–F). The P/G@1BG group exhibited the highest BV/TV (59.85 ± 7.39%) and relative BMD (8.00 ± 0.40-fold relative to control), accompanied by increased Tb.N (2.87 ± 0.63-fold) and reduced Tb.Sp (0.56 ± 0.07-fold). These findings indicate that the P/G@1BG hydrogels efficiently promote the formation of dense, interconnected trabecular bone and facilitate the structural reconstruction of cranial bone defects.

**Fig. 6:**
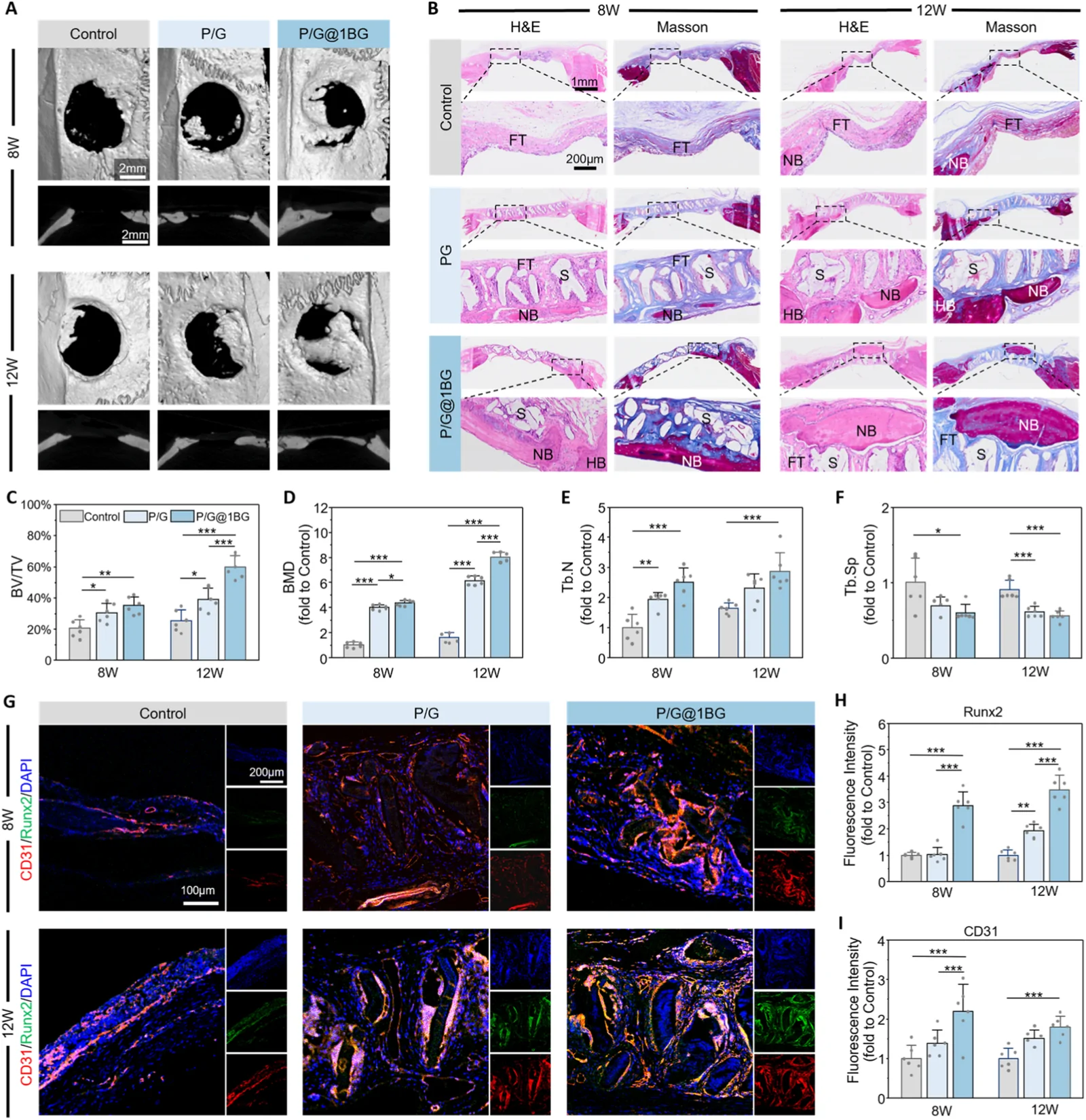
*In vivo* osteogenic and angiogenic evaluation using a rat calvarial defect model. (A) Micro-CT 3D reconstruction and coronal sectional images at 8 and 12 weeks. (B) H&E and Masson's trichrome staining at 8 and 12 weeks (HB: host bone, NB: new bone, FT: fibrous tissue, S: residual scaffold). (C–F) Quantitative analysis of BV/TV, BMD, Tb.N, and Tb.Sp at 8 and 12 weeks. (G) Double immunofluorescence staining for Runx2 and CD31 at 8 and 12 weeks. (H–I) Semi-quantitative analysis of Runx2 and CD31 fluorescence intensity. *p < 0.05, **p < 0.01, ***p < 0.001.

H&E staining was performed to characterize the histological morphology of regenerated tissues (Fig. 6B). Blank defects were predominantly filled with loose fibrous tissue, with limited new bone confined to the defect edges. The P/G hydrogel merely maintained defect space and induced marginal osteogenesis. All PLGA-based composite scaffolds underwent gradual *in vivo* degradation accompanied by host tissue ingrowth, achieving desirable scaffold remodeling and tissue integration, which is consistent with previous studies [41]. Notably, the P/G@1BG group significantly enhanced new bone formation with tight integration between neo-bone and host bone. Only mild inflammatory infiltration was observed across all groups, confirming the satisfactory histocompatibility of fabricated hydrogels.

Masson's trichrome staining was applied to assess collagen deposition and bone maturation (Fig. 6B). At week 8, blank defects showed sparse collagen deposition and minimal osteogenesis, and only limited bone regeneration occurred around P/G scaffolds. In contrast, the P/G@1BG group exhibited more continuous and mature bone tissue formation within the defect region. At week 12, tissues within blank defects remained poorly mineralized and structurally disordered, whereas the P/G@1BG hydrogel guided the ordered formation of lamellar bone rich in mature collagen, with newly formed bone closely deposited along scaffold surfaces. These results confirm that P/G@1BG hydrogel exerts favorable osteoconductivity and effectively accelerates bone mineralization and maturation.

To elucidate the interplay between osteogenesis and angiogenesis, immunohistochemical staining of the osteogenic marker Runx2 and endothelial marker CD31 was conducted (Fig. S11). At 12 weeks post-implantation, the relative expression levels of Runx2 and CD31 in the P/G@1BG group were 3.0-fold and 2.8-fold higher than those in the blank group, respectively. Double immunofluorescence staining at defect sites further corroborated this synergistic effect (Fig. 6G–I). The blank group showed faint Runx2 and CD31 fluorescence signals, and the P/G group displayed moderate signal intensity. In comparison, the P/G@1BG group exhibited the strongest and most widely distributed fluorescence signals. Persistent colocalization of Runx2-positive osteoblasts and CD31-positive neovessels was observed at both 8 and 12 weeks, demonstrating that *in vivo* osteogenesis and angiogenesis occur in a spatiotemporally coupled manner rather than proceeding independently.

In summary, these *in vivo* findings fully validate the rationale and advantages of the P/G@1BG composite hydrogel design. The MEW-fabricated PLGA scaffold maintains stable defect morphology and provides structural guidance for tissue ingrowth, achieving mechanical–biological decoupling via rigid physical support rather than excessive hydrogel crosslinking. The degradable GelMA/BG matrix undergoes in situ dynamic remodeling to construct a bioactive microenvironment that activates osteogenic differentiation and facilitates vascular ingrowth. This functional division endows the rigid skeleton with mechanical bearing capacity while allowing the hydrogel component to dominate biological regulation. Such elaborate design successfully decouples mechanical stability from biological activity, coordinates coupled osteogenic and angiogenic events, and ultimately facilitates the regeneration of high-quality vascularized bone tissue.

### 3.8 Transcriptomic analysis of osteogenic–angiogenic responses

To elucidate the molecular basis of P/G@1BG-mediated osteogenic–angiogenic coupling, transcriptome sequencing was performed on rBMSCs cultured with composite hydrogels. Volcano plots and heatmaps revealed evident transcriptional reprogramming in the P/G@1BG group, with 784 significantly upregulated differentially expressed genes (DEGs) and 401 downregulated DEGs compared with the control group (Fig. 7A and B). Gene Ontology (GO) enrichment analysis of upregulated DEGs across biological processes, cellular components, and molecular functions indicated that P/G@1BG hydrogels fostered a favorable regenerative microenvironment by promoting rBMSCs proliferation and extracellular matrix deposition (Fig. 7C). Kyoto Encyclopedia of Genes and Genomes (KEGG) pathway enrichment analysis further identified the Ras/MAPK signaling pathway among the significantly enriched pathways in rBMSCs cultured with P/G@1BG hydrogels (Fig. 7D), suggesting its potential involvement in the cellular response to the composite hydrogel.

**Fig. 7:**
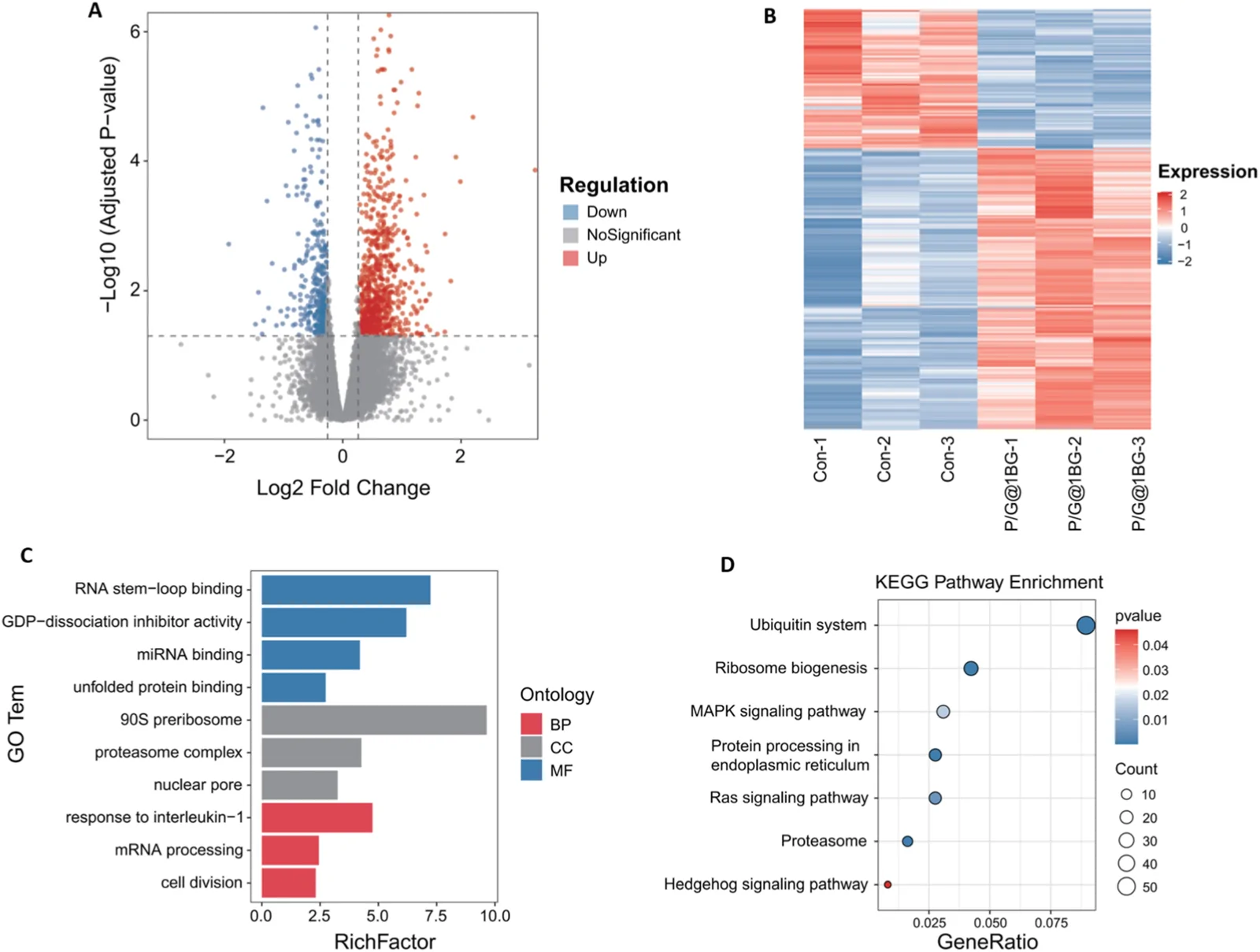
RNA transcriptomic analysis of rBMSCs cultured on P/G@1BG hydrogels. (A) Volcano plot of DEGs between the P/G@1BG and blank control groups. (B) Hierarchical clustering heatmap of DEGs. (C) GO enrichment analysis of upregulated DEGs (BP: biological process, CC: cellular component, MF: molecular function). (D) KEGG pathway enrichment analysis of upregulated DEGs.

In conjunction with the qRT-PCR-verified upregulation of HIF-1α and VEGFA (Fig. 4H and I), the enrichment of Ras/MAPK signaling suggests a potential bifunctional role in coordinating osteogenic and angiogenic programs. In the osteogenic branch, microenvironmental stimuli are transduced through the Ras/MAPK axis to activate Runx2-mediated transcriptional cascades, upregulating osteogenic markers including ALP, OPN and OCN, thereby driving rBMSCs osteogenic commitment, matrix maturation, and biomineralization [53]. In the angiogenic branch, Ras/MAPK signaling functions as an upstream regulator that elevates the expression of downstream HIF-1α and VEGFA. This cascade further initiates paracrine pro-angiogenic responses that facilitate endothelial cell recruitment, migration, and capillary network formation [54]. Specifically, rBMSCs stimulated by P/G@1BG hydrogels secrete abundant VEGFA, which supports endothelial cell adhesion, proliferation and functional activation [55]. In return, newly formed blood vessels supply nutrients and oxygen to sustain stable maturation of regenerated bone tissue [56]. Collectively, these findings suggest that Ras/MAPK-related signaling may contribute to the coordinated osteogenic and pro-angiogenic responses induced by P/G@1BG hydrogels. However, further studies incorporating additional scaffold control groups and functional perturbation of the Ras/MAPK pathway will be required to clarify component-specific transcriptional responses and establish pathway causality.

## 4 Conclusion

In conclusion, this study develops a bioresorbable composite hydrogel by integrating bioactive glass functionalized GelMA matrix within MEW-structured PLGA microfibrous scaffolds for craniofacial bone reconstruction. The optimized P/G@1BG composite hydrogel exhibits tunable degradation behavior, favorable mechanical properties, excellent cytocompatibility, and sustained release of bioactive ions. Transcriptomic analysis further identified enrichment of the Ras/MAPK signaling pathway, suggesting its potential involvement in the osteogenic and pro-angiogenic responses induced by the composite hydrogel. *In vivo* animal studies further confirm that this scaffold induces robust vascularized neo-bone formation at 12 weeks post-implantation.

This structurally synergistic design achieves effective separation of mechanical and biological performances, overcoming the inherent functional trade-off that commonly limits conventional hydrogel platforms for bone repair. By dissociating mechanical structural support from bioactive microenvironment regulation, the hydrogel not only overcomes the traditional material design challenges but also significantly enhances cooperative interplay between bone-forming and vascularization behaviors. This work demonstrates that rationally decoupling conflicting material properties can precisely orchestrate synergistic regenerative outcomes, offering a promising design paradigm for advanced craniofacial bone repair.

## CRediT authorship contribution statement

**Yue Hong:** Writing – original draft, Validation, Methodology, Investigation, Formal analysis, Data curation. **Huali Lu:** Writing – original draft, Validation, Methodology, Investigation, Formal analysis, Data curation. **Jie Luo:** Validation, Investigation, Formal analysis, Data curation. **Yaoyi Li:** Validation, Investigation, Formal analysis, Data curation. **Yizi Ye:** Validation, Investigation, Formal analysis, Data curation. **Yuning Hua:** Validation, Investigation, Formal analysis, Data curation. **Huaizhong Xu:** Writing – review & editing, Supervision, Resources, Project administration, Funding acquisition, Conceptualization. **Ting Xu:** Writing – review & editing, Supervision, Resources, Project administration, Funding acquisition, Conceptualization.

## Funding

This work was supported by the 10.13039/501100001809National Natural Science Foundation of China (Grant No. 62177040), the 10.13039/501100001691Japan Society for the Promotion of Science (JSPS KAKENHI, Grant No. JP24K17184), and the Fund of 10.13039/501100004543China Scholarship Council (202308330108).

## Declaration of competing interests

The authors declare that they have no known competing financial interests or personal relationships that could have appeared to influence the work reported in this paper.

## Data Availability

Data will be made available on request.
