# Supplementary material for: Synergistically reinforced bioresorbable hydrogel for mechanical–biological decoupling and osteogenic–angiogenic coupling
Source: Mater Today Bio. 2026 Oct 13;41:103736. doi: 10.1016/j.mtbio.2026.103736 (PMC13698973; doi:10.1016/j.mtbio.2026.103736)
Supplement: Supplementary file 1 [file mmc1.docx]

Supporting Information

**Synergistically Reinforced Bioresorbable Hydrogel for Mechanical–Biological Decoupling and Osteogenic–Angiogenic Coupling**

*Yue Hong^1,#^, Huali Lu^2,#^, Jie Luo^1^, Yaoyi Li^1^, Yizi Ye^1^, Yuning Hua^1^, Huaizhong Xu^2,^*, Ting Xu^1,^**

^1^ Department of Stomatology, The First Affiliated Hospital, Zhejiang University School of Medicine, Hangzhou 310003, P. R. China

^2^ Department of Biobased Materials Science, Kyoto Institute of Technology, Matsugasaki, Kyoto 606-8585, Japan

*Corresponding author

E-mail:

xhz2008@kit.ac.jp (H. Xu, ORCID: 0000-0001-7291-1936)

xut@zju.edu.cn (T. Xu, ORCID: 0000-0003-3086-6916)

\# Y. Hong and H. Lu contributed equally to this work.

**Supplementary Figures**

**Figure S1**. Optical and SEM images of 50-layered PLGA scaffolds fabricated via MEW with three distinct porous geometries. The fabrication was conducted under the following optimized parameters: a melting temperature of 170 °C, a collector temperature of 75 °C, a nitrogen pressure of 0.12 MPa, a nozzle-to-collector distance of 2.5 mm, an applied voltage of 4.0 kV, and a printing speed of 7 mm s^-1^.

**Figure S2**. Water contact angle (WCA) measurements of PLGA scaffolds before and after surface modification. (A) Untreated PLGA scaffold displaying a hydrophobic surface with a WCA of 116.9°. (B) Hydrophilized PLGA scaffold showcasing complete wetting after surface treatment with 0.1 M NaOH solution for 4.5 h.

**Figure S3**. Microstructural morphology of composite hydrogels with different BG contents. Representative SEM images of freeze-dried P/G, P/G@1BG, P/G@5BG, and P/G@10BG composite hydrogels. All groups exhibited interconnected porous hydrogel networks embedded within the ordered PLGA microfibrous scaffolds, without obvious interfacial separation between the PLGA fibers and the surrounding GelMA matrix.

**Figure S4**. Cumulative ion release from P/G@BG hydrogels. Time-dependent cumulative concentrations of (A) P ions and (B) Si ions released from P/G@BG scaffolds (1BG, 5BG, and 10BG) in deionized water at 37 °C for up to 21 days. The concentrations were quantified via inductively coupled plasma mass spectrometry (ICP-MS) from the extracts collected at predetermined intervals.

**Figure S5**. GPC chromatograms of PLGA and PCL scaffolds during accelerated degradation in NaOH solution. Normalized RI curves as a function of elution time for (A) PLGA scaffolds over 72 h and (B) PCL scaffolds over 7 d, showing a progressive shift of the peaks toward longer retention times, indicative of molecular weight reduction.

**Figure S6**. ALP staining and semi-quantitative analysis of rBMSCs cultured with different hydrogels extracts for 14 days. (A) Representative images of ALP staining in the control, P/G, P/G@1BG, and P/G@5BG groups. (B) Quantitative analysis of the ALP-positive area, showing the highest osteogenic activity in the P/G@1BG group. **p* < 0.05, ***p* < 0.01.

**Figure S7**. ALP staining and semi-quantitative analysis of rBMSCs cultured on different hydrogels. (A) Representative images of ALP staining in rBMSCs seeded on P/G, P/G@1BG, and P/G@5BG hydrogels after 7 and 14 days of culture. (B) Quantification of the ALP-positive area, demonstrating enhanced osteogenic differentiation in the BG-containing groups. At day 14, the ALP-positive areas in the P/G@1BG and P/G@5BG groups were significantly greater than those in the P/G group. **p* < 0.05, ***p* < 0.01, ****p* < 0.001.

**Figure S8**. ARS staining and semi-quantitative analysis of mineralization nodules formation in rBMSCs cultured on different hydrogels. (A) Representative images of ARS staining in rBMSCs seeded on P/G, P/G@1BG, and P/G@5BG hydrogels after 21 days of osteogenic induction. (B) Quantification of the ARS-positive area, showing significantly greater calcium deposition in the P/G@1BG and P/G@5BG groups than in the P/G group. ***p* < 0.01, ****p* < 0.001.

**Figure S9**. qRT-PCR analysis of osteogenic gene expression in rBMSCs after 7 and 14 days of culture. Relative mRNA expression levels of (A) BMP-2, (B) Runx2, and (C) COL1A1 were quantified in the control, P/G, P/G@1BG, and P/G@5BG groups. The P/G@1BG group consistently exhibited the highest expression of all three osteogenic genes at both time points. **p* < 0.05, ***p* < 0.01, ****p* < 0.001.

**Figure S10**. Histological evaluation of major organs after hydrogel implantation. Representative H&E-stained sections of the heart, liver, spleen, lung, and kidney from the control, P/G, and P/G@1BG groups are shown. No obvious inflammatory infiltration, tissue necrosis, or other histopathological abnormalities were observed in any group, indicating favorable systemic biosafety of the composite hydrogels.

**Figure S11.** Immunohistochemical evaluation of osteogenesis and angiogenesis at 8 and 12 weeks post-implantation. (A) Representative immunohistochemical staining images of Runx2 and CD31 in the control, P/G, and P/G@1BG groups. Black arrows indicate positively stained regions. (B, C) Semi-quantitative analysis of Runx2- and CD31-positive areas, respectively, normalized to the control group. The P/G@1BG group exhibited markedly enhanced Runx2 and CD31 expression, particularly at 12 weeks.
